# Comprehensiveness of distributed medical education systems: a regional population-based perspective

**DOI:** 10.1186/s12909-020-02466-x

**Published:** 2021-01-09

**Authors:** Andrea M Burrows, Kevin B Laupland

**Affiliations:** 1 Research and Knowledge Translation, Royal Inland Hospital and Interior Health Authority, 311 Columbia Street, British Columbia V2C 2T1 Kamloops, Canada; 2 Queensland University of Technology (QUT), Queensland Brisbane, Australia; 3 Department of Intensive Care Services, Royal Brisbane and Women’s Hospital, Queensland Brisbane, Australia

**Keywords:** Residency training program, Distributed medical education, Undergraduate medical students, Academic faculty

## Abstract

**Background:**

The core business of medical schools includes clinical (education and service) and academic (research) activities. Our objective was to assess the degree to which these activities exist in a distributed medical education system in Canada.

**Methods:**

A population-based design was utilized. Programs were contacted and public records were searched for medical trainees and faculty positions within a province in Canada during the 2017/2018 academic year. Data were expressed as positions per 100,000 residents within the Lower Mainland, Island, and Northern and Southern interior geographical regions.

**Results:**

Substantial differences in the distribution of medical students by region was observed with the highest observed in the Northern region at 45.5 per 100,000 as compared to Lower Mainland, Island, and Southern regions of 25.4, 16.8, 16.0 per 100,000, respectively. The distribution of family medicine residents was less variable with 14.9, 10.7, 8.9, and 5.8 per 100,000 in the Northern, Island, Southern, and Lower Mainland regions, respectively. In contrast, there was a marked disparity in distribution of specialty residents with 40.8 per 100,000 in the Lower Mainland as compared to 7.5, 3.2, and 1.3 per 100,000 in the Island, Northern, and Southern regions, respectively. Clinical faculty were distributed with the highest observed in the Northern region at 180.4 per 100,000 as compared to Southern, Island, and Lower Mainland regions of 166.9, 138.5, and 128.4, respectively. In contrast, academic faculty were disproportionately represented in the Lower Mainland and Island regions (92.8 and 50.7 per 100,000) as compared to the Northern and Southern (1.4 and 1.2 per 100,000) regions, respectively.

**Conclusions:**

While there has been successful redistribution of medical students, family medicine residents, and clinical faculty, this has not been the case for specialty residents and academic faculty.

## Background

Traditionally, medical education has been centralized in large urban centers with the majority of the core business conducted in tertiary or quaternary institutions [1]. However, it has been increasingly recognized that such urban-centric medical training models have failed to adequately address the needs of the population with underservicing of smaller cities and communities [2–5]. In response, distributed medical education systems have been implemented worldwide and the evolution of the decentralization of medical education is well documented in Canada, Australia, USA, and South Africa [1, 6–11]. In a distributed education system, medical schools are organized within a hub and spoke structure with a parent program and “satellite” sites; these satellite sites are located in cities and where trainees perform most of their training external to the main parent program [1, 8, 12]. The diversification of training contexts has evolved to address disparity in physician location of practice, health inequalities among populations by developing competencies tailored to community needs and enhancing social diversity of medical student population [6, 11]. There is limited research comparing the distribution of faculty and trainees across the medical school structure and the implications in education, clinical service and research this may have for learners, faculty and communities.

It is widely accepted that the core business of medical schools is to provide undergraduate and graduate medical education, clinical service, and conduct research [5, 11, 13, 14]. In order to provide opportunities for both trainees as well as faculty, and to benefit the populations they serve, it follows that these core activities be distributed across the medical school structure. The objective of this study was to evaluate the geographical distribution of medical trainees and faculty within a decentralized medical education system in Canada.

## Methods

**Fig. 1:**
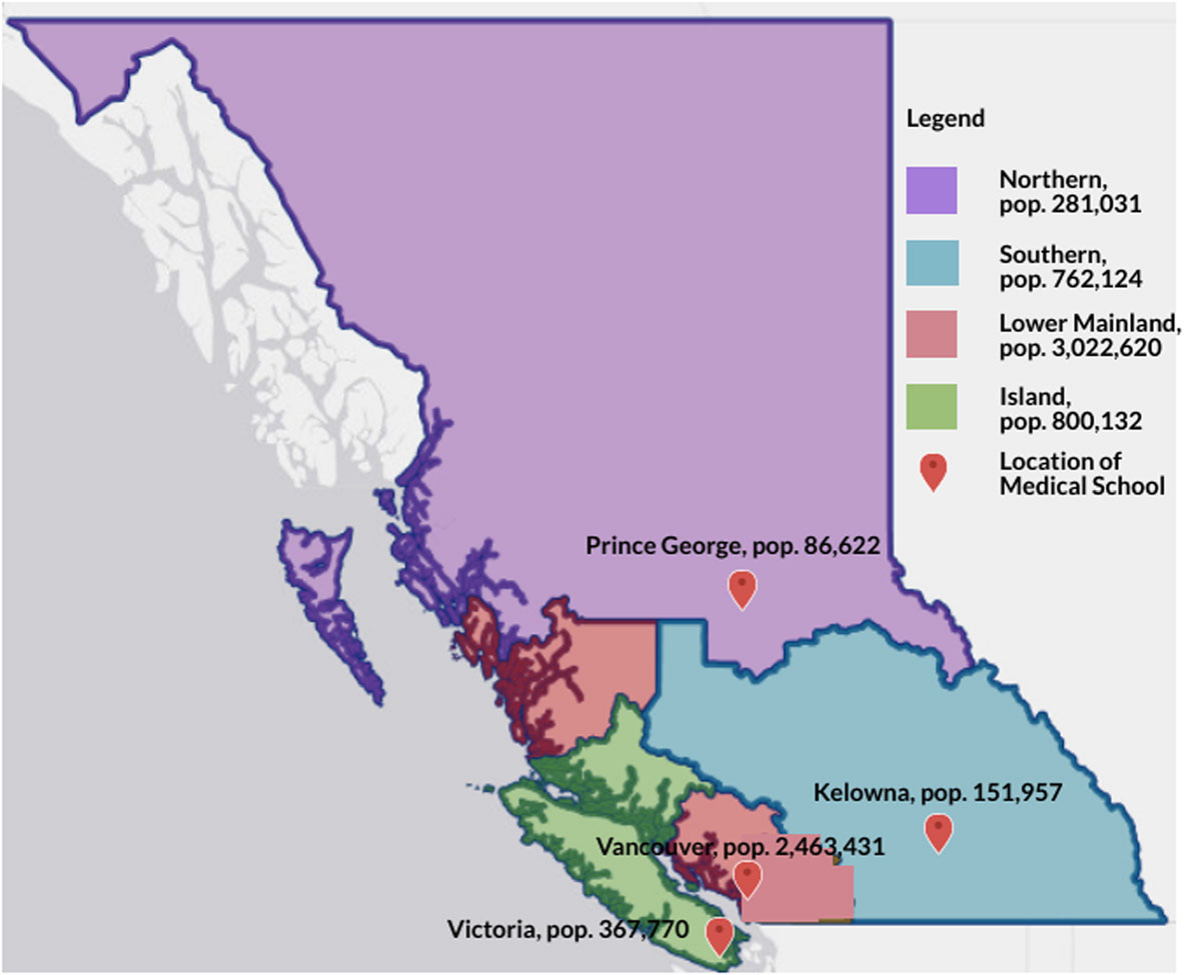
The location of medical schools within their respective health region boundaries in British Columbia, Canada

The study design was a population-based comparative cohort. We sub-classified the population of British Columbia (4,865,907) into regions corresponding to the catchment areas of medical programs, regional health authorities that they primarily serve and geographical area (Fig. 1). These included the Lower Mainland (Vancouver/Fraser/Providence; population 3,022,620; area 67,081 km^2^), Island (Vancouver Island Health Authority; population 800,132; area 84,232 km^2^), and Northern (Northern Health; population 281,031; area 172,523 km^2^), and Southern (Interior Health; population 762,124; area 215,422 km^2^) regions [15, 16]. Given that this study was a review of publicly available aggregate data, we did not require ethics approval.

Within the four regions we established faculty and trainee numbers using one or more of: published data, web-based information, or personal contact with program administrators and/or UBC Access and Privacy. The 2017/2018 academic year was chosen. The Vancouver-Fraser, Island, Southern, and Northern Medical Programs were attributed to their respective regions for medical students [17]. Family medicine and specialty residents were classified to region based on the primary placement location within health region boundaries [18]. Family medicine residency positions included registered Canadian and international medical graduates. Clinical and academic faculty were similarly classified or as allocated to universities based within these regions.

We *a priori* specified that the following would be collected as measures of faculty and trainees: number of undergraduate doctor of medicine (MD) program students per year; number of family medicine and specialty resident trainees per year; clinical faculty (i.e., Clinical Assistant/Associate/Professor, Clinical Instructor); and, academic faculty (Associate/Assistant/Professor) [17]. Given that there are possibilities for trainees to perform training in multiple regions (i.e., electives) and for educators to be cross-appointed in multiple areas, we classified trainees and educators based on their primary affiliation or parent program for analysis. For comparative analysis data were reported as rates per 100,000 population based on health authority census data available from BC statistics [16].

## Results

Satellite sites are located in cities while the parent program is located in large urban center. There are marked differences in population density by health region with the highest observed in the Lower Mainland with 45 people per square km as compared to Island, Interior and Northern of 9, 4, 2 respectively. In the year 2017/2018, there were a total 1152 medical students, 359 family medicine residents, and 1321 specialty residents registered, and 9993 clinical and academic faculty identified.

Among the total number of medical students (1152), there were 288 per year of the four-year program. Within each year one third (96/288) trained outside of the Lower Mainland with 32 students at each of the three distributed sites. There were marked differences in the distribution of medical students by region adjusted by population with the highest observed in the Northern region at 45.5 per 100,000 as compared to Lower Mainland, Island, and Southern regions of 25.4, 16.8, 16.00 per 100,000, respectively.

**Fig. 2:**
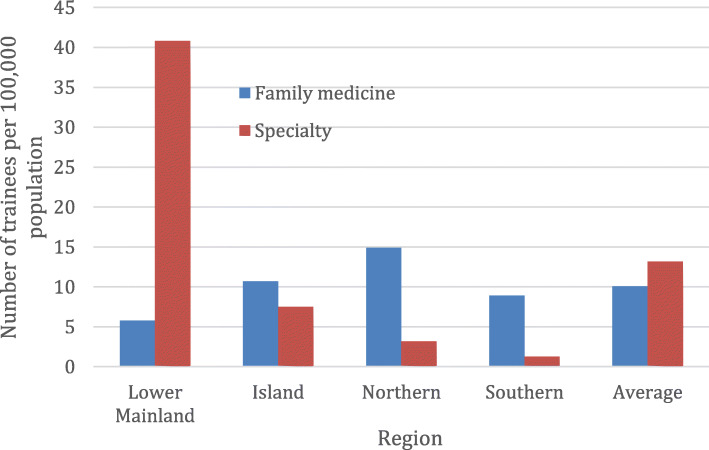
Distribution of family medicine and specialty resident trainees by region in British Columbia, 2017/2018

As shown in Fig. 2, there was a highly unequal distribution of resident trainees across the four regions of the province. The combined number of residents adjusted per population in the Lower Mainland (46.6 per 100,000) was more than twice each of the Island (18.2 per 100,000) and Northern (18.1 per 100,000) regions and almost five times that of the Southern (10.2 per 100,000) region (Fig. 2).

Each year there are 174 family medicine entry positions for the two-year program. In 2017/18, there were a total of 359 family medicine residents and half were trained outside of the Lower Mainland region. There were marked differences in distribution for family medicine residents with highest observed rates in Northern region at 14.9 per 100,000 as compared to Island, Southern and Lower Mainland regions of 10.7, 8.9 and 5.8 per 100,0000, respectively (Fig. 2). In contrast, the distribution of specialty residents in Lower Mainland region at 40.8 per 100,000 as compared to Island, Northern and Southern regions of 7.5, 3.2 and 1.3 per 100,000 respectively (Fig. 2). Notably, among the total number of specialty residents, only 6% (79/1312) were trained primarily outside the Lower Mainland region.

**Fig. 3:**
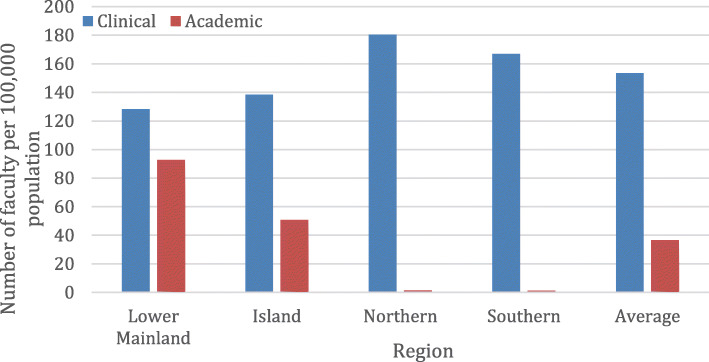
Distribution of clinical and academic medical faculty by region in British Columbia, 2017/2018

There were marked differences in distribution of medical faculty across regions as shown in Fig. 3. In absolute numbers clinical faculty (*n* = 9401) account for the majority (94%) of the appointments and were distributed across the regions. There was relatively limited variability with the highest observed in Northern at 180.4 per 100,000 as compared to Southern, Island and Lower Mainland regions of 166.9, 138.5, and 128.4, respectively (Fig. 3). In contrast, academic faculty varied dramatically by region with rates in the Lower Mainland and Island regions of 92.8 and 50.7 per 100,000 as compared to 1.4 and 1.2 per 100,000 in the Northern and Southern regions, respectively (Fig. 3).

## Discussion

The decentralization of medical education has distributed some aspects of the medical school structure; however, specialty resident trainees and academic faculty are disproportionately concentrated in the Lower Mainland and Island regions. In a distributed medical education system, undergraduate medical students and family medicine resident trainees are distributed across three satellite sites. Clinical faculty make up the majority (94%) of the workforce and make significant contributions to the teaching of medical students and family medicine residents. In contrast, the concentration of academic faculty (95%) and specialty resident trainees (94%) are observed in the large urban center of the Lower Mainland. Failing to distribute some of the core activities of service, research and education in the medical school structure consequently may create disadvantages for learners, faculty and limit opportunities for patients to benefit from specialist services and research advances.

Distributed medical education systems, where medical trainees conduct much or most of their training in smaller satellite or branch sites of a large traditional medical school has evolved in several countries in order to address the limitations of the traditional model [12, 19–21]. Previous work has focused on the distribution of undergraduate medical education and family medicine programs and finds positive outcomes in experiences and perceptions of learners in regional and rural sites, benefits to communities and development of competencies tailored to community needs [2, 7–9, 19, 22–24]. A number of important social and economic benefits related to enrichment of the medical community by a distributed system have been identified, including increasing social diversity in medical education [2, 22, 25, 26]. However, there continues to be underrepresentation from Indigenous Peoples, people of lower socioeconomic status and from rural backgrounds in medical education [8, 9, 19, 27–30]. They face multiple barriers as prospective students, including, financial, travel and academic preparation to support admission [28, 29].

The shortage of family practice physicians in smaller communities has propelled distributed medical education systems forward; and the establishment of longitudinal rural and regional campuses have led to physician migration from large urban centers, addressing some shortages in smaller communities [8, 9, 19, 21, 22, 25, 26, 31–33]. This is evident in British Columbia; since 2004, the University of British Columbia, Faculty of Medicine implemented 3 satellite sites and distributed undergraduate and family medicine residency training. The successful distribution of practicing family physicians in 2018 is observed in the small variation across regions, with highest on Island at 176.6 per 100,000 as compared to Northern, Southern and Lower Mainland of 155.1, 150.2, and 122.7, respectively [34].

Physician specialists are, however, underrepresented in smaller communities and there is little evidence of specialty residency training programs and/or academic faculty distribution. The discipline-specific rotations in smaller communities continues to be used for specialty residency programs and although clinical rotations are perceived to be valuable and increase self-identified likelihood of regional practice, there are limited data to indicate any shift in interest towards living in a smaller site [23, 35, 36]. For example, in the distributed model in British Columbia, only 6% (79/1312) of subspecialty residents are based outside the Lower Mainland, despite, specialty services being provided across all regions. This follows observed differences in practicing specialty physicians across British Columbia with highest observed in Lower Mainland and Island at 130.8 and 117.5 per 100,000, as compared to Southern and Northern, at 97.6 and 63.0, respectively [34].

In a distributed medical education system, human resources would be allocated to match the clinical and academic infrastructure. Clinical faculty make up the majority of the workforce and volunteer to supervise and teach learners in the clinical setting. For these faculty, teaching is tied to clinical revenue and the distribution of medical students is labour intensive, can decrease productivity and limit the number of trainees in a community [37–39]. In contrast, as observed in the findings, academic faculty predominately reside in a densely populated urbanized area and supervise the majority of resident trainees. For academic faculty, these trainees may significantly decrease workload and provide more time to conduct research and compete for national funding. Senior residents, for example, allocate a quarter of their time teaching interns and medical students; and they act as mentors, providing leadership, guidance and learning [13]. With few residency programs, there may be fewer opportunities for residents to teach at distributed sites as compared to their urban colleagues [38]. It helps that medical students are distributed to increase opportunities for teaching, however, urban residents participate in a broad community consistent with multiple learners, and more opportunities to supervise and teach while working as a team in the context of clinical practice [2, 38].

An equal balance between service and medical education may be indicated by resident research [14]. Residents that undertake research are competitive for subspecialty fellowships or careers in academia. Active participation in research exposes residents to scientific methods, improves critical appraisal of literature, nurtures critical thinking and generates new medical knowledge [13]. The medical community can then support environments of inquiry and scholarship in which residents participate in the development of new knowledge, evaluate research findings and translate knowledge at the bedside [14]. However, major barriers exist that limit resident participation in research outside urban centers. Few distributed specialty residency programs provide less opportunity for clinicians to be exposed to research, develop research skills, and grow specialty careers; and clinical faculty are at a disadvantage to conduct and support resident research because they have inadequate protected time, less research training, access to granting agencies, office and administrative support. There are well documented impediments to research activity in residency training programs, including, time, interest, qualified mentors and cost; however, trained faculty and their involvement have shown to increase research activity [39–41]. Research training is identified as important for clinicians to conduct research and mentor trainees; however, research productivity and development is tied to protected time, research career development and mentorship [39, 40]. The participation in research sets medical education apart from other health professional training. Research attracts funding, improves patient care and influences career pathways. Research in tertiary large centers often lacks real world application, and residents that participate and conduct research during their fellowship are more likely to devote working time and future careers to clinical research [39]. The redistribution of residency programs and academic faculty will support research programs that address population health needs.

### Limitations

The secondary analysis of existing data describes the current state of medical education at a single point in time and may include a certain level of bias due to absence of statistical tests. Our results were collected from cross linking data from multiple publicly available sources. While we studied one distributed medical system and used it as an example to demonstrate the issue, the findings justify the evaluation of other distributed systems.

## Conclusions

In a distributed medical education system, the core activities of medical schools including education, service and research are spread out across a geographical area. From a comprehensive, population-based perspective, the findings demonstrate a distributed system failing to provide some of the core activities of medical school structure and justify the need for further evaluation of distributed systems. The distribution of undergraduate and family medicine residency programs helps address critical shortages of health professionals in smaller communities. However, few specialty residency programs and academic faculty outside urban centers does not meet population health needs and are barriers to advancements in healthcare and specialty careers. Major redistribution of specialty resident trainees and academic faculty positions are needed to result in a truly distributed medical education system.

## Data Availability

The data generated or analysed during this study are included in this published article.
